# Comparison of Gene Editing Versus Conventional Breeding to Introgress the *POLLED* Allele Into the Tropically Adapted Australian Beef Cattle Population

**DOI:** 10.3389/fgene.2021.593154

**Published:** 2021-02-11

**Authors:** Maci L. Mueller, John B. Cole, Natalie K. Connors, David J. Johnston, Imtiaz A. S. Randhawa, Alison L. Van Eenennaam

**Affiliations:** ^1^Department of Animal Science, University of California, Davis, Davis, CA, United States; ^2^Animal Genomics and Improvement Laboratory, Agricultural Research Service, United States Department of Agricultural, Beltsville, MD, United States; ^3^Animal Genetics and Breeding Unit (AGBU), University of New England, Armidale, NSW, Australia; ^4^School of Veterinary Science, The University of Queensland, Gatton, QLD, Australia

**Keywords:** gene editing, beef cattle, Brahman, Australia, simulation, polled

## Abstract

Dehorning is the process of physically removing horns to protect animals and humans from injury, but the process is costly, unpleasant, and faces increasing public scrutiny. Genetic selection for polled (hornless), which is genetically dominant to horned, is a long-term solution to eliminate the need for dehorning. However, due to the limited number of polled Australian Brahman bulls, the northern Australian beef cattle population remains predominantly horned. The potential to use gene editing to produce high-genetic-merit polled cattle was recently demonstrated. To further explore the concept, this study simulated introgression of the *POLLED* allele into a tropically adapted Australian beef cattle population via conventional breeding or gene editing (top 1% or 10% of seedstock bulls/year) for 3 polled mating schemes and compared results to baseline selection on genetic merit (Japan Ox selection index, $JapOx) alone, over the course of 20 years. The baseline scenario did not significantly decrease the 20-year *HORNED* allele frequency (80%), but resulted in one of the fastest rates of genetic gain ($8.00/year). Compared to the baseline, the conventional breeding scenarios where polled bulls were preferentially used for breeding, regardless of their genetic merit, significantly decreased the 20-year *HORNED* allele frequency (30%), but resulted in a significantly slower rate of genetic gain ($6.70/year, *P* ≤ 0.05). The mating scheme that required the exclusive use of homozygous polled bulls, resulted in the lowest 20-year *HORNED* allele frequency (8%), but this conventional breeding scenario resulted in the slowest rate of genetic gain ($5.50/year). The addition of gene editing the top 1% or 10% of seedstock bull calves/year to each conventional breeding scenario resulted in significantly faster rates of genetic gain (up to $8.10/year, *P* ≤ 0.05). Overall, our study demonstrates that, due to the limited number of polled Australian Brahman bulls, strong selection pressure on polled will be necessary to meaningfully increase the number of polled animals in this population. Moreover, these scenarios illustrate how gene editing could be a tool for accelerating the development of high-genetic-merit homozygous polled sires to mitigate the current trade-off of slower genetic gain associated with decreasing *HORNED* allele frequency in the Australian Brahman population.

## Introduction

Intact horns are extremely costly due to carcass bruising, hide damage, and danger to human handlers (Commonwealth Scientific and Industrial Research Organisation [CSIRO], 2014). Horn removal, or dehorning, is therefore a standard cattle management practice. However, the process is expensive, painful for the animal, and subject to increasing public scrutiny. It is recommended to dehorn cattle at as early of an age as possible because the costs and invasiveness associated with the procedure, and the attendant animal welfare concerns, increase as the animal grows older.

There are two different methods to remove horns depending on the age of the calf, disbudding and dehorning. In cattle, horns first form as a free-floating horn bud, usually evident at birth. If a calf is less than 8 weeks old, the unattached horn buds can be removed by disbudding via a hot iron or caustic paste. However, after this time frame the growing horn attaches to the skull and must be physically removed by dehorning using a scoop, gouge, saw, or wire. Amputating the fixed horn from the skull can expose the frontal sinuses, which is part of the cranial cavity that houses the brain, and typically results in larger and more invasive wounds (Weaver et al., 2018). These wounds take longer to heal and increase the chance of infection, especially in warmer climates, often leading to short-term weight loss (Winks et al., 1977).

### Dehorning Practices in Northern Australia

Over 170,000 calves are dehorned each year in Australia and almost 100,000 of those are in northern Australia (Queensland, the Northern Territory, and northern Western Australia) (Animal Health Australia [AHA], 2014b). In northern Australia, beef cattle production is based on extensive grazing systems and mating seasons are typically ill-defined so calves can range in age between 3.5 and 10 months at the time of first handling (Bortolussi et al., 2005). Moreover, any calves that miss this first processing will have an even longer dehorning delay. A study of factors associated with calf mortality in this system found that almost all calf deaths post-branding occurred in calves that were dehorned, totaling 2.1% of all dehorned calves (Bunter et al., 2013).

In addition to the economic costs, common livestock management practices, such as dehorning, are under increasing public scrutiny (Ventura et al., 2015). In response to mounting consumer concerns, countries are implementing improved animal welfare policies and practices (Stafford and Mellor, 2011). Current Animal Welfare Standards enforced by Animal Health Australia (AHA), prohibit dehorning without the use of appropriate pain relief, unless the animal is (1) less than 6 months old, or (2) less than 12 months old if it is the animal’s first time being yarded or brought into a pen/enclosure. The age limit is also dependent on individual State or Territory jurisdictions (Animal Health Australia [AHA], 2014a). Appropriate pain relief is defined as, “the administration of drugs that reduce the intensity and duration of a pain response” (Animal Health Australia [AHA], 2014a). It should be noted that dehorning cattle is a requirement of the Live Export Accreditation Program (LEAP), which states that slaughter and feeder cattle shall not be exported unless they are polled or dehorned, and each horn stump is less than 12 cm in length and fully healed (LiveCorp-Australia, 2001).

Moreover, dehorning in Australia is likely to be subject to stricter animal welfare legislation in the future (Animal Health Australia [AHA], 2014b). In 2014 when the Animal Welfare Standards were being revised, AHA held an open public consultation period and received over 1,500 responses. The most controversial issue was related to “pain relief for surgical procedures (e.g., castration and dehorning).” Recommendations received included suggestions to require pain relief irrespective of the age of the animal, and some even suggested a complete ban of all dehorning practices.

### Dehorning Alternative: *POLLED* Genetics

An alternative to dehorning is to use genetic selection to increase the number of polled (hornless) cattle. Horns are inherited as an autosomal recessive trait (Long and Gregory, 1978). To date, four candidate *POLLED* mutations have been identified in *Bos taurus* cattle on chromosome 1 (BTA1). The most common is a simple allele of Celtic origin (P_*C*_) corresponding to a duplication of a 212 bp sequence in place of a 10-bp deletion on chromosome 1 (Medugorac et al., 2012). Another allele is an 80,128-bp duplication of Friesian origin (P_F_; Rothammer et al., 2014). A third mutation, a complex 219-bp duplication–insertion (P_219ID_), and a 7-bp deletion and 6-bp insertion (P_1ID_) was identified through admixed Mongolian yaks (Medugorac et al., 2017). A fourth allele (P_*G*_), an approximately 110 kb duplication was revealed by genome-wide association studies of Nelore (*Bos taurus indicus*) beef cattle (Stafuzza et al., 2018; Utsunomiya et al., 2019).

Several studies have reported dehorning practices and investigated the *POLLED* allele frequency of dairy cattle populations (Gottardo et al., 2011; Cozzi et al., 2015; Götz et al., 2015). An extensive survey was done in Europe on dehorning methods and the frequency of polledness in different breeds (Cozzi et al., 2015). Data showed that in Europe, 81% of dairy, 47% of beef and 68% of suckler farms physically disbudded or dehorned animals. Only 7.3% of the beef farms reported keeping polled cattle. This is a surprisingly low number given the availability of the P_*C*_ allele in sires in a number of beef cattle breeds. Beef and suckler farms were found to have a higher prevalence of dehorning cattle older than 2 months, which is more invasive compared to disbudding methods commonly used in the dairy industry.

There have been attempts to increase *POLLED* allele frequency in dairy breeds through systematic breeding. For example, the Fleckvieh dual purpose strain started to select for polled with the introduction of a single founder cow in 1974. Prior to 1990 the *POLLED* allele had been common only in beef strains of this breed, but not in the dual-purpose strain, because the available polled bulls had very low breeding values for milk yield. The number of polled bulls available for artificial insemination (AI) has since increased considerably through systematic breeding, genetic testing, and the introduction of genomic breeding values in late August 2011. As a result, the proportion of polled cows in the Fleckvieh dual-purpose population is expected to be around 10.5% by 2021 (Götz et al., 2015).

Similarly, in the Netherlands selection for polled Holstein’s has been enhanced by genomic selection, and this has enabled the selection of polled bulls with improved genetic merit (NVI-Dutch Flemish Index) such that they’re only about 5 years of genomic selection behind elite horned bulls (Windig et al., 2015). However, homozygous polled Holstein bulls currently available in the Netherlands have a relatively high inbreeding level as they all originated from the same two founder polled bulls.

On the other hand, there has been less of a need for investigating and modeling polled beef breeding schemes due to the availability of efficient beef producing genetics in homozygous polled beef breeds, like Angus. For example, in the United States there was a 58% reduction in beef calves born with horns from 1992 to 2007 (United States Department of Agriculture [USDA], 2008) and in EU member states only 47% of beef producers report dehorning compared to 81% of dairy producers (Cozzi et al., 2015). Although artificial selection and crossbreeding with polled beef breeds has successfully been used to increase the number of polled beef animals in temperate climates, like the United States and Europe, these options have not been as successful in tropical climates, such as northern Australia (Prayaga, 2007; Connors et al., 2018; Lyons and Randhawa, 2020).

Brahman, the primary breed used in northern Australia beef production systems due to their parasite resistance and tropical climate adaptability, are predominantly horned. However, a recent genome sequencing study in Australian Brahmans provides evidence that the *POLLED* mutation present in this breed is the P_*C*_ allele, and that it has been introgressed from *Bos taurus* cattle (Koufariotis et al., 2018). Other genetic factors (i.e., scur and African horn) have also been associated with the presence/absence of horns. However, these factors are believed to segregate independently from the *POLLED* locus and are influenced by the animal’s sex (Long and Gregory, 1978; Asai et al., 2004; Wiedemar et al., 2014).

Animal health organizations in Australia strongly recommend breeding for polled cattle whenever possible (Newman, 2007; Animal Health Australia [AHA], 2014a) and several *POLLED* genetic tests have been developed (Connors et al., 2018). Most recently, an “optimized poll gene test” was developed that can successfully predict the genotype of 99% of samples assessed, including *B. indicus*-influenced breeds (Lyons and Randhawa, 2020; Randhawa et al., 2020). Despite the development of *POLLED* diagnostic tests, increasing the frequency of *POLLED* through conventional breeding alone has been challenging due to the limited number of polled Brahman sires (Prayaga, 2007; Lyons and Randhawa, 2020).

### Potential to Use Gene Editing to Produce High-Genetic-Merit Polled Sires

An additional strategy that has been proposed to decrease the need for dehorning, is the use of gene editing to produce high-genetic-merit polled sires. Gene editing refers to the use of site-directed nucleases to precisely introduce a double stranded break at a specific location in the genome. In 2016, gene editing was used to achieve an intraspecies P_*C*_ allele introgression in cattle (212-bp duplication replacing a 10-bp sequence on BTA1) to produce two healthy, homozygous polled dairy bulls (Carlson et al., 2016).

Our previous simulation study modeling the United States dairy cattle population (Mueller et al., 2019) and a simulation study modeling a generic livestock population (Bastiaansen et al., 2018) both found that the use of gene editing was the most effective way to decrease the frequency of recessive alleles (e.g., *HORNED*), while minimizing detrimental effects on inbreeding and genetic merit. In these simulation studies, gene editing reduced the undesired allele significantly faster than genomic selection or conventional breeding strategies alone. Additionally, both studies found that gene editing reduced long-term inbreeding levels in scenarios that placed all selection emphasis on the monogenic trait. Furthermore, these studies found that the addition of gene editing helped minimize the decrease in the rate of genetic gain resulting from selection emphasis on a monogenic trait.

However, there are several unique economic and translational considerations of extending gene editing for the *POLLED* allele to the beef industry as compared to the dairy industry. For instance, the majority of dairy calves are produced via artificial insemination (AI) (VanRaden, 2007), whereas beef calves are primarily the result of natural service matings (Husted, 2018). Additionally, in temperate production systems with early dehorning (e.g., United States and European dairy industries), mortality rates from dehorning are generally considered to be almost negligible (Seppä-Lassila et al., 2016; Winder et al., 2018). In contrast, dehorning was found to be an important calf mortality factor in a study investigating tropically adapted beef breeds managed in extensive Australian production systems (Bunter et al., 2013).

**FIGURE 1:**
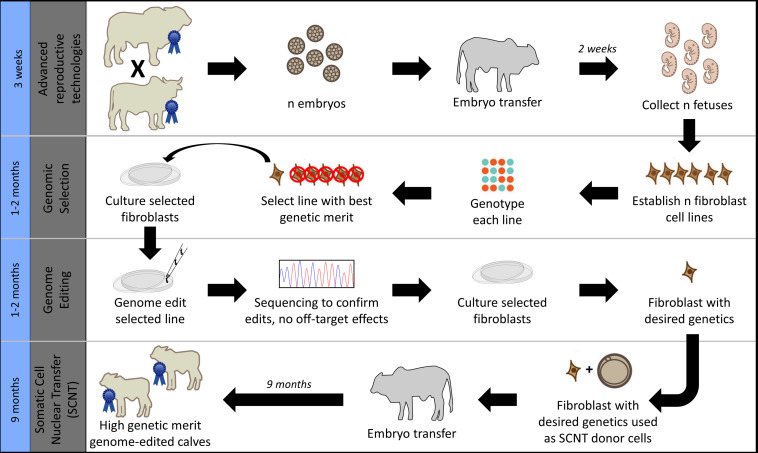
Gene editing was modeled as an added step to the elite sire production system proposed by Kasinathan et al. (2015), which combines the use of advanced reproductive technologies and somatic cell nuclear transfer cloning (SCNT) with embryo transfer (ET). Image modified from Van Eenennaam (2017) used with permission.

Given these industry differences and the large number of beef calves currently being dehorned in northern Australia, this study aimed to investigate strategies for increasing *POLLED* allele frequency in the northern Australian Brahman beef cattle population. In particular, we sought to compare current conventional breeding/selection strategies used in this population with the addition of gene editing. Additionally, we compared and contrasted the results of this beef population simulation to our previous United States dairy simulation results (Mueller et al., 2019), as both were modeled using the same gene editing strategy of an added step to the elite sire production system proposed by Kasinathan et al. (2015) (Figure 1).

The objectives of this study were to (1) estimate the availability and genetic merit of polled Brahman bulls available in Australia, and (2) simulate introgression of the *POLLED* allele into the northern Australian Brahman beef cattle population via conventional breeding or gene editing for multiple polled mating schemes as compared to a baseline scenario of selection on genetic merit alone using the Japan Ox selection index ($JapOx), over the course of 20 years.

## Materials and Methods

Computer simulation using the program “geneedit” (Cole and Mueller, 2019) was used to compare introgression of the *POLLED* allele into the Australian Brahman population via conventional breeding or with the additional use of gene editing.

### Simulation Program

The “geneedit” program simulates gene editing applied to a cattle population as an extension of Cole’s program (2015) to manage multiple recessives. Complete source code is available on GitHub and is in the public domain. The basic simulation procedures are identical to those described in detail by Cole (2015), with extensions added to support gene editing, permit more flexible selection of polled sires, and model a population with nucleus and multiplier herds.

First, base bull and cow populations are formed by simulating animals with varying ages by drawing true breeding values (TBV) from normal distributions and randomly sampling horned genotypes from a Bernoulli distribution with a parameter equal to its allele frequency in the population of interest. The program ensures that at least one carrier bull and cow will be present in the base population for each recessive. Also, to prevent a minor allele from being lost due to drift a constant mutation rate of 10^–5^ is used, which results in “AA” genotypes being converted to “Aa” genotypes when calves are created. All base population animals are treated as founders and mated at random for 10 years to produce the population used for specific mating schemes.

Each round of the simulation represents 1 year of calendar time, and generations overlap. The population size increases each round of the simulation until the user defined maximum population size of bulls and cows is reached. Each year, animals are culled for reaching the user defined maximum permitted age. Also, if the population is too large after age-based culling then cows will be culled at random (involuntary culls) and bulls with the lowest TBV or other user defined culling criteria will be culled.

Any deviations from the basic simulation procedures described above, the specific mating schemes and gene editing strategy modeled in this Australian Brahman simulation study are described in detail in the section below and complete simulation parameters are listed in the Appendix Table 1.

The program is a stochastic simulation, but individual replicates can be recreated if the same seed is used for random variate generation. When the software was designed the goal was to examine changes in allele frequencies in cattle populations under different mating strategies, not to create a comprehensive general-purpose simulator for animal breeding. Because of that focus, selection is on TBV that are assumed known without error rather than estimated breeding values (EBV). This also means that genomic selection is not supported.

### Modeling the Australian Brahman Beef Cattle Population

A population of 10 nucleus (seedstock) herds supplying breeding bulls to 200 multiplier (commercial) herds was modeled. Ten replicates of each scenario were simulated for 20 years, with overlapping generations.

All simulations were performed on a Thinkmate RAX QS6-4210 (Thinkmate, Inc., Waltham, MA, United States) workstation with four 12-core AMD Opteron 6344 processors with a clock speed of 2.6 GHz, 512 GB of DDR3 1600 MHz RAM, and CentOS Linux EL7.

#### Estimating the Availability and Genetic Merit of Polled Australian Brahman Beef Cattle

*POLLED* allele frequency and the number of homozygous polled, heterozygous polled and horned sires were calculated from Australian Brahman animals with a SNP-based *POLLED* test (GeneSeek^®^) results from 2014 to 2017 (Lyons and Randhawa, 2020). It should be noted that only the genotypic data at the *POLLED* locus was available on these bulls. Additionally, the average EBV of the standard Australian Brahman economic selection index, Japan Ox ($JapOx), was calculated for each sire group as an indicator of overall genetic merit (Australian Brahman EBVs, 2018, unpublished data).

The GeneSeek^®^ SNP-based *POLLED* test data used to derive allele frequency and genetic merit estimates in this study may not be completely representative of the Australian Brahman population because the *POLLED* test is bundled with parentage and higher-density SNP genotyping for the Australian Brahman single-step genomic evaluation. There may have been a non-random selection of phenotypically polled animals submitted to determine if they were homozygous polled. However, this dataset included a large number of herds and different sires, so estimates based on this dataset were used for further modeling. All values are in Australian dollars. Two-tailed, unpaired student *t*-tests were used to determine if the average $JapOx value for each genotype was significantly different from one another. *P*-values of ≤0.05 were considered to be significantly different.

#### Northern Australia Beef Production Practices

Northern Australia is a vast region, covering approximately 400 million ha located within the subtropics and tropics. Major characteristics of this production system are low stocking rates (up to 1 animal unit per 150 ha), large management groups (500–1,000 animals), multi-sire matings, and irregular handling and husbandry of cattle—typically only twice annually (Bortolussi et al., 2005; Burns et al., 2010). In large part due to the extensive management system, it is estimated that AI is used by less than 1% of northern Australian breeding herds (Meat and Livestock Australia Limited [MLA], 2015).

Due to the challenging tropical environment, approximately 85% of northern Australian cattle are *B. indicus* and *B. indicus* cross, primarily through the use of Brahman cattle (Bortolussi et al., 2005). Several northern Australia studies, reviewed by Burns et al. (2010), have demonstrated that tropically adapted heifers do not reach puberty as yearlings, but the majority will by 24 months of age. Therefore, heifers are typically first joined to calve as 3-year-olds and re-joined annually unless culled. Calf losses to branding or weaning are considerable, ranging between 4 and 31% across cow populations of mixed age, breed and a range of production environments (Burns et al., 2010).

#### Simulation Base Population Structure

In this simulation, the seedstock base population was 15,000 cows and 600 bulls. The commercial base population was 35,000 cows and 1,500 bulls. These unrelated base population animals were assigned a birth year from −10 to 0 (cows) or −5 to 0 (bulls) by sampling from a uniform distribution. True breeding values ($JapOx) for each animal were determined by randomly sampling from a normal distribution, with a standard deviation (SD) of $34 for both the seedstock and commercial populations, and a mean of $34 for seedstock cows and $0 for commercial cows (Johnston and Graser, 2009). Base population bulls averaged one genetic SD higher than cows, $68 and $34 for seedstock and commercial bulls, respectively. Each base population animal’s horned status was determined by randomly sampling sire and dam alleles using a *HORNED* allele frequency of 80% (Lyons and Randhawa, 2020). Moreover, the proportion of polled bulls in the base population was set to 30% heterozygous and 2.6% homozygous. Additionally, homozygous polled bulls in the base population averaged 0.16 SD ($5.44) lower $JapOx than horned bulls (Australian Brahman EBVs, 2018, unpublished data).

#### Simulation Scheme

**FIGURE 2:**
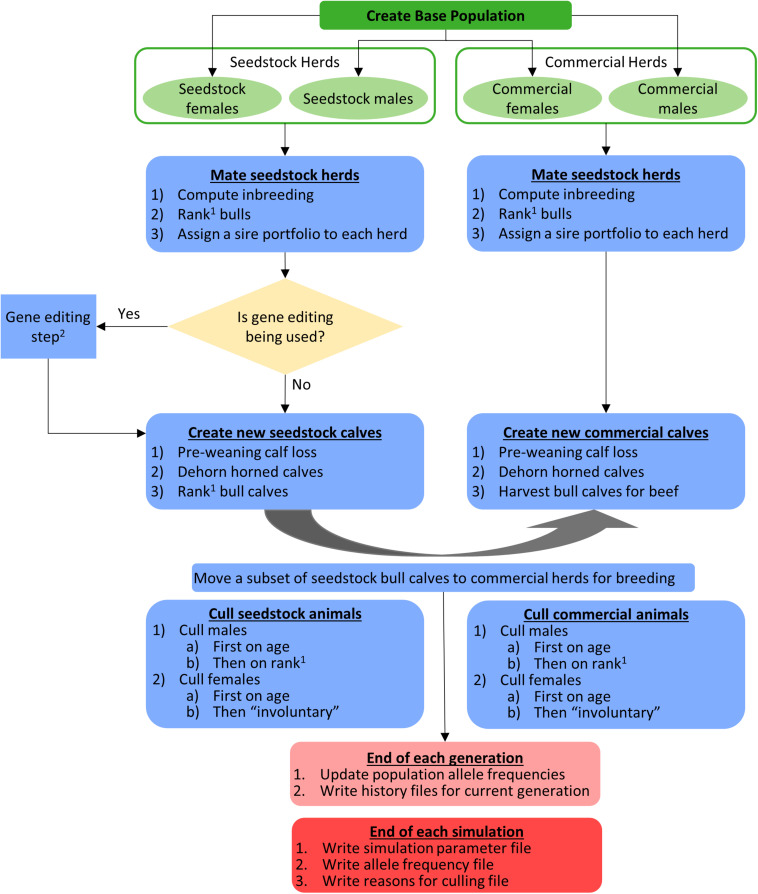
Schematic of the flow of operations in the simulation. ^1^The order of bull ranking was based on true breeding values (TBV) of the Japan Ox selection index ($JapOx) and, depending on the mating scheme used, also by polled genotype. ^2^See Figures 1, 3 for a detailed gene editing model and flowchart, respectively.

The flow of operations in the simulation is shown schematically in Figure 2. The different mating schemes, and when applicable, gene editing, started in year 0. Females were mated to have their first calf at age 3 and bulls were eligible for breeding at age 2. Mating via natural service was modeled so each herd used a unique portfolio of bulls, bulls within a sire portfolio were mated randomly to cows in the herd, and each bull was limited to 35 matings per year. The polled/horned genotype and source of bulls (seedstock or commercial) depended on the mating scheme used (described below). Females did not move between herds.

Calves were born annually in the same herd as their dams. Sex was assigned randomly to new calves with a 50:50 sex ratio. The TBV for new calves were created by taking the parent average and adding a Mendelian sampling term (MS): TBV_calf_ = 0.5(TBV_sire_ + TBV_dam_) + MS, where TBV_calf_, TBV_sire_, and TBV_dam_ are the TBV of the calf, its sire, and its dam, respectively. The Mendelian sampling term was drawn from a normal distribution with a mean of 0 and a variance of $\frac{1}{2}\,\left[1-\frac{1}{2}\,\left(f_{\text{S}}+f_{\text{D}}\right)\right]\,\sigma_{\text{a}}^{2},$ where *f*_*S*_ and *f*_*D*_ are the coefficients of inbreeding of the sire and dam, respectively, and $\sigma_{a}^{2}$ is the additive genetic variance of $JapOx (Cole, 2015). Inbreeding was estimated without error based on pedigree relationships (Aguilar and Misztal, 2008) using the program INBUPGF90 version 1.42 (Aguilar and Misztal, 2012). All base population animals were assumed to be unrelated, which is improbable in a commercial setting as polled bulls are likely to have common lineages. To determine a new calf’s horned status, one allele was sampled at random from each parent and used to construct the progeny genotype. Recessive genotypes were simulated without error, and it was only necessary to simulate genotypes for recessive alleles because pedigrees were assumed to be free of errors. Allele frequencies were updated each year by counting alleles (Cole, 2015).

**FIGURE 3:**
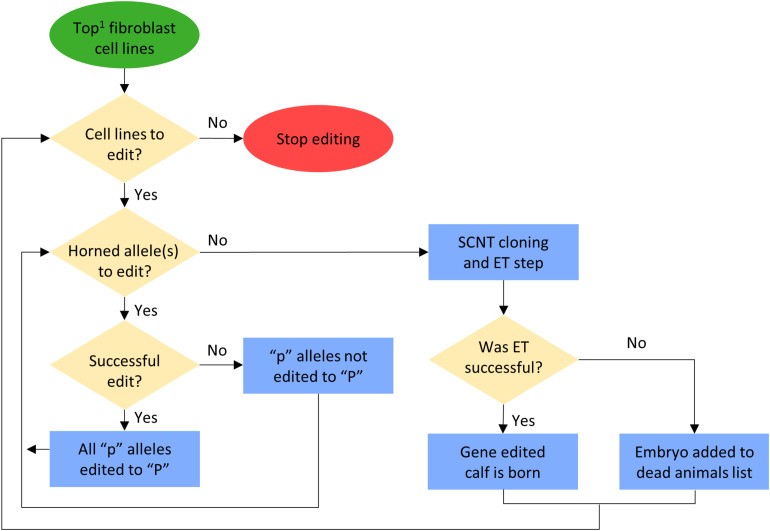
Flowchart for gene editing and embryo transfer (ET) process in the simulation. ^1^Potential calves were sorted by their true breeding values (TBV) for the Japan Ox selection index ($JapOx) (highest to lowest).

In scenarios that included gene editing (Table 1), it was modeled as an added step to the elite sire production system proposed by Kasinathan et al. (2015). In this system, fetal tissue from the next generation of yet-to-be born bulls is genomically screened and selected, edited, and then somatic cell nuclear transfer (SCNT) cloning is used to create embryos for embryo transfer (ET) (Figure 1). In our simulation, both heterozygous polled and horned seedstock male fetuses were sorted on $JapOx and the top 1% or 10% were gene edited to be homozygous polled and then cloned to produce a live calf. The gene editing technology modeled had editing and ET success rates of 61 and 21%, respectively. In order for a gene edited calf to be born both the edit and the ET had to be successful (Figure 3). The “geneedit” program allows the user to set a maximum number of attempts for editing and ET. However, as we modeled gene editing of a fetal cell line, which allowed for continuous editing attempts and confirmation of a successful edit before SCNT cloning and ET, the editing and ET processes were repeated until a successful outcome was observed. Under this idealized system no costs or time lag to achieve an edited bull were factored into the gene editing scenarios. In reality, there would be obvious logistical and economic considerations that would need to be evaluated to determine feasibility of gene editing to achieve homozygous polled bulls. As outlined in Figure 1, in practice the proposed gene editing system is anticipated to require an additional 3–5 months to produce a gene edited, homozygous polled bull.

**TABLE 1:** Parameters and results of each scenario.

Mating scheme	1^*o*^ bull selection criterion	Scenario	Gene edit^3^	20-year *HORNED* allele frequency (%)		20-year Inbreeding (%)		20-year $JapOx^1^	
				Seedstock	Commercial	Seedstock	Commercial	Seedstock	Commercial
A	$JapOx^1^	A	0%	^*a*^78.0 ± 0.7	^*a*^78.0 ± 0.6	^*a*^0.70 ± 0.01	^*a*^0.005 ± 0.0	^*a*^226 ± 0.8	^*a*^160 ± 0.6
B	Homozygous and heterozygous polled	B	0%	^*b*^8.9 ± 0.2	^*cb*^29.4 ± 0.2	^*a*^0.70 ± 0.02	^*a*^0.005 ± 0.0	^*d*^194 ± 1.5	^*d*^133 ± 1.2
		B_1%	1%	^*b*^8.9 ± 0.1	^*b*^29.5 ± 0.2	^*b*^0.85 ± 0.02	^*a*^0.005 ± 0.0	^*b*^216 ± 0.7	^*b*^150 ± 0.5
		B_10%	10%	^*b*^8.8 ± 0.1	^*cd*^28.2 ± 0.1	^*ab*^0.71 ± 0.01	^*a*^0.005 ± 0.0	^*a*^225 ± 0.6	^*a*^160 ± 0.5
C	Homozygous polled	C	0%	^*b*^8.3 ± 0.1	^*cb*^28.9 ± 0.1	^*ab*^0.76 ± 0.03	^*a*^0.006 ± 0.0	^*c*^198 ± 0.8	^*c*^137 ± 0.6
		C_1%	1%	^*b*^8.5 ± 0.2	^*cb*^28.7 ± 0.2	^*b*^0.85 ± 0.03	^*a*^0.005 ± 0.0	^*b*^214 ± 0.6	^*b*^151 ± 0.4
		C_10%	10%	^*b*^8.2 ± 0.2	^*d*^27.4 ± 0.2	^*ab*^0.71 ± 0.02	^*a*^0.005 ± 0.0	^*a*^227 ± 1.0	^*a*^162 ± 0.7
D	Homozygous polled_only^2^	D	0%	^*c*^6.6 ± 0.1	^*e*^8.00 ± 0.0	_*ab*_0.73 ± 0.02	^*b*^0.011 ± 0.0	^f^178 ± 0.9	^f^110 ± 0.7
		D_1%	1%	^*c*^6.1 ± 0.1	^*e*^8.00 ± 0.0	_*ab*_0.75 ± 0.03	^*b*^0.012 ± 0.0	^*e*^188 ± 1.4	^*e*^118 ± 0.7
		D_10%	10%	^*c*^6.2 ± 0.1	^*e*^8.10 ± 0.0	^*ab*^0.72 ± 0.05	^*b*^0.012 ± 0.0	^*d*^193 ± 1.0	^*d*^125 ± 0.9

*^1^Japan Ox selection index.*

*^2^Scenario D only allowed the use of homozygous polled bulls for breeding.*

*^3^In the gene editing scenarios, the top 1% or 10% of horned or heterozygous polled seedstock bull calves per year were gene edited to be homozygous polled.*

*^*a*–*f*^Means within a column with different superscripts differ (P ≤ 0.05).*

New calves were subject randomly to a pre-weaning calf loss of 8% (seedstock) or 13% (commercial). Additionally, new horned calves of both populations were subject randomly to a dehorning mortality rate of 2% (Bunter et al., 2013). Removal of scurs, which are sometimes mistaken for horns and occur in some heterozygous polled animals, could also decrease calf survival rate, but was not considered in this simulation.

Seedstock bull calves were kept for 1 year and then as yearling bulls they were sorted by $JapOx ranking (highest to lowest) and, depending on the mating scheme used, also by polled genotype. The seedstock population retained the top 5% of their yearling bulls (as defined by the mating scheme) for breeding to seedstock cows and the remainder of seedstock yearling bulls were moved to the commercial population for mating to commercial cows. In contrast, commercial population bull calves were all castrated and sold for beef annually, unless noted otherwise in a mating scheme.

Animals were culled at the end of a simulated year. In both populations, cows were culled first by age (≥10 years) and then in order to not exceed a maximum populations size of 3,000 seedstock (∼1,800 breeding age) and 100,000 commercial (∼61,000 breeding age) females, they were also culled through random selection, which modeled involuntary culling. Bulls were culled first by age (≥5 years, i.e., after 3 years of breeding). Additionally, to maintain the allowable population size bulls were culled by the lowest $JapOx ranking and variably by polled genotype, depending on the mating scheme used, [culling order: (1) horned, (2) heterozygous polled, (3) homozygous polled]. The maximum allowable population sizes were 60 seedstock and 2,000 commercial mating-age-bulls (i.e., ≥2 years old).

#### Simulation Mating Schemes

Four total mating schemes, one baseline (A), two polled preference (B and C) and one obligatory polled (D) were modeled (Table 1). The three polled mating schemes (B, C, and D) were modeled using conventional breeding methods alone, and the addition of gene editing the top 1 and 10% of seedstock bull claves per year, for a total of 10 scenarios.

For retention in the seedstock population, baseline scheme A ranked the seedstock yearling bulls solely on $JapOx. Whereas polled mating schemes (B, C, and D) ranked the seedstock yearling bulls first on polled genotype [retaining order: (1) homozygous polled, (2) heterozygous polled, (3) horned] and then on $JapOx. In baseline scheme (A) and polled preference schemes (B and C), all bulls used for breeding were sourced from the seedstock population.

To establish a baseline and model current practice, scheme A used $JapOx as the sole sire selection criterion (i.e., all polled/horned sire genotypes could be used for breeding).

In polled preference scheme B, polled bulls (both heterozygous and homozygous) were preferentially used for breeding, regardless of their genetic merit ($JapOx). If the maximum number of matings was reached for all polled bulls before all females were covered, horned bulls were used to cover the remaining females. Polled preference scheme C preferentially used only homozygous polled bulls for breeding and if needed, both heterozygous polled and horned seedstock bulls were used to cover the remaining females.

In contrast, obligatory polled scheme (D) could only use homozygous polled bulls for breeding. If there were not enough seedstock homozygous polled bulls, then homozygous polled bulls from the commercial population were used to fill the deficit. As previously mentioned, the genetic merit ($JapOx) of these commercial bulls was on average one standard deviation less ($34) than seedstock bulls ($68). This scheme models what might be expected if producers are legally or contractually prohibited from using genetics that result in horned offspring.

#### Simulation Data Analysis and Visualization

**FIGURE 4:**
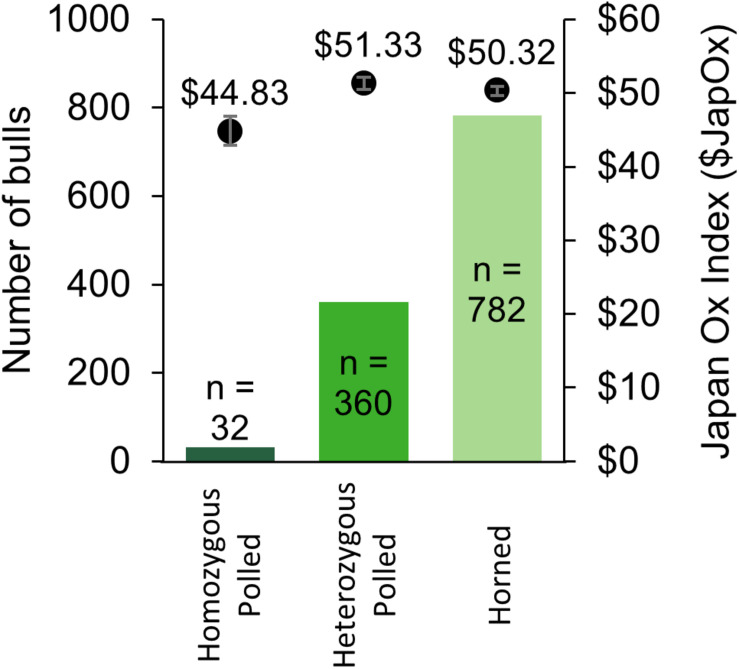
Summary of Australian Brahman bulls with a SNP-based *POLLED* test (GeneSeek^®^) result from 2014 to 2017. The number of bulls per genotype is on the primary *y*-axis (solid bars) and the average Japan Ox selection index ($JapOx) value of the top 20% of bulls per genotype is on the secondary *y*-axis (dots with error bars representing SEM).

**FIGURE 5:**
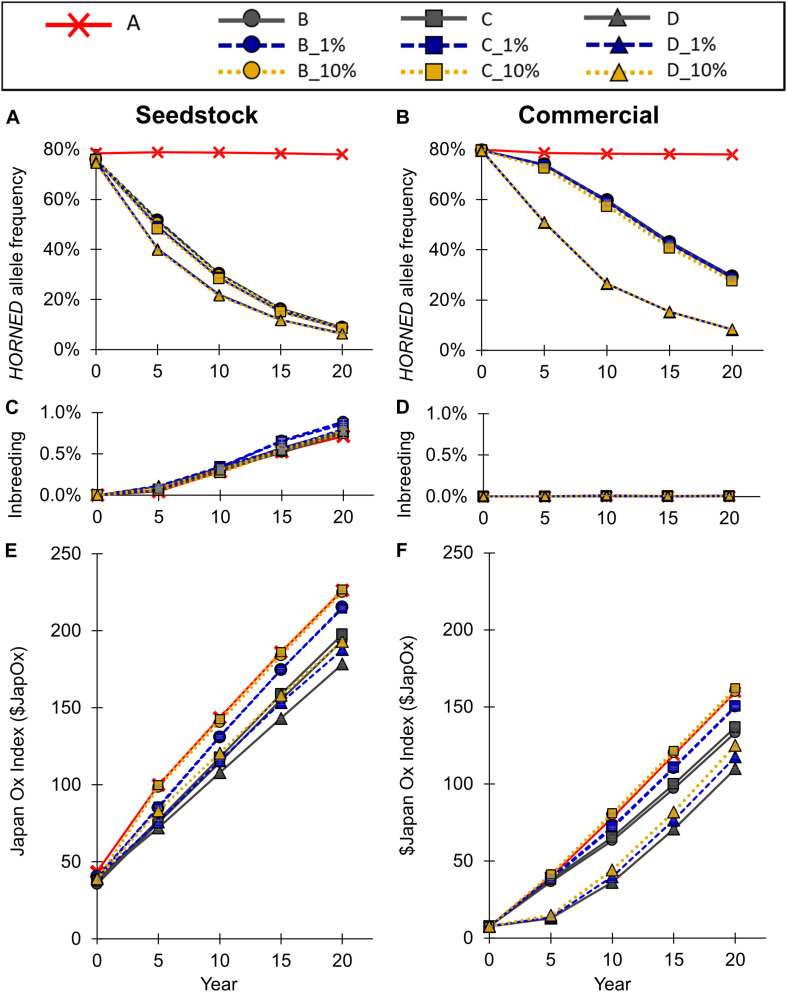
Effect of each scenario on **(A,B)** *HORNED* allele frequency, **(B,C)** inbreeding, and **(E,F)** genetic merit for the seedstock **(A,C,E)** and commercial **(B,D,F)** populations. Conventional breeding scenarios (A,B,C,D) are solid lines, gene editing 1% scenarios (B_1%, C_1%, D_1%) are dashed lines, and gene editing 10% scenarios (B_10%, C_10%, D_10%) are dotted lines. Error bars represent SEM.

**FIGURE 6:**
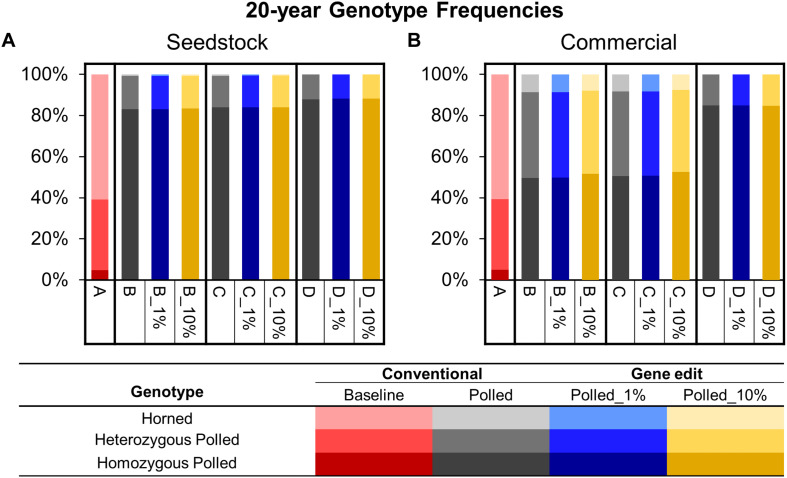
Estimated genotype frequencies in year 20 of each scenario for the seedstock **(A)** and commercial **(B)** populations. Genotypes are represented by a color scale from darkest (homozygous polled) to lightest (horned).

Each scenario was replicated 10 times using a different seed for the random number generator. The actual values used were saved to an output file so that results could be replicated if necessary. The changes in *HORNED* allele frequency, inbreeding, $JapOx, the total number of animals sold for beef per year, and the number and genotype of sires used per year were compared between all 10 scenarios. Additionally, Hardy–Weinberg principles were used to estimate the 20-year genotype frequencies based on the calculated *HORNED* allele frequencies of each scenario. Significance of the changes in *HORNED* allele frequency, inbreeding levels, the total number of animals sold for beef per year, and the number and genotype of sires used per year were determined using Tukey’s Honest Significant Difference test. To determine significance of changes in the rate of genetic gain, linear regressions of TBV for $JapOx on birth year were used. *P*-values of ≤0.05 were considered to be significantly different for all analyses. The results (Figures 3–6) presented are the means of the 10 replicates for each scenario and the variation between the replicates is represented by standard error of the mean bars.

## Results

### Availability and Genetic Merit of Polled Bulls

From 2014 to 2017 there were a total of 1,533 Australian Brahman animals from 22 different herds with a SNP-based *POLLED* test (GeneSeek^®^) result (“Australian Brahman data,” 2018). The results consisted of 39 homozygous polled, 443 heterozygous polled, and 1,051 horned animals. The allele frequency was calculated to be 20% for the current Australian Brahman population. The 39 homozygous polled animals were sired by 25 different sires (not including unknown sires). Out of the tested animals, 1,174 were male and consisted of 32 homozygous polled, 360 heterozygous polled and 782 horned (Figure 4). The observed frequencies were not significantly different from those expected under Hardy–Weinberg equilibrium. The average $JapOx value of the top 20% of horned, heterozygous polled and homozygous polled males were $50.32, $51.33 (*P* = 0.32 compared to horned) and $44.83 (*P* ≤ 0.05 compared to horned), respectively (Figure 4).

### Seedstock Population

#### *HORNED* Allele Frequency

To model the current Australian Brahman population, all scenarios started with a *HORNED* allele frequency of 80% (Figure 5A). In baseline scenario A, which placed no selection pressure on polled, the seedstock population *HORNED* allele frequency remained near 80% throughout all 20 years. Consequently, after 20 years of baseline scenario A the seedstock population was estimated to consist of only 5% homozygous polled, 34% heterozygous polled and a majority (61%) of horned animals (Figure 6A). In contrast, selection of polled sires in the conventional breeding scenarios (B, C, and D) decreased the 20-year *HORNED* allele frequency by 69.1% (95% CI [68%, 70%]), 69.7% (95% CI [68%, 71%]), and 71.4% (95% CI [70%, 73%]), respectively (*P* ≤ 0.05 compared to A; Figure 5A).

The 20-year *HORNED* allele frequencies of polled preference conventional breeding scenarios, B and C, were 8.9% (95% CI [8.4% 8.9%]) and 8.3% (95% CI [7.8%, 8.8%], *P* ≥ 0.05 compared to B), respectively (Figure 5B). As a result, after 20 years both polled preference scenario B and C’s commercial populations were estimated to be ∼83% homozygous polled and only ∼1% horned (Figure 6A). The addition of gene editing the top 1 and 10% of seedstock bull calves per year (B_1% and B_10%; C_1% and C_10%) similarly decreased the 20-year *HORNED* allele frequency (*P* ≥ 0.9 compared to B and C, respectively).

The obligatory polled scheme D resulted in the lowest 20-year *HORNED* allele frequency of 6% (95% CI [5.6%, 7.1%]). In scheme D only homozygous polled sires were used, thus conventional breeding and the addition of gene editing the top 1 and 10% of seedstock bull calves per year resulted in the same (*P* ≥ 0.9 compared to D) rapid decrease in *HORNED* allele frequency (Figure 5A). Additionally, scheme D was estimated to result in the highest percentage of homozygous polled (87%) and 0 horned animals in year 20 (Figure 6B).

#### Inbreeding

The seedstock population inbreeding levels inbreeding remained below 1% in all scenarios (Figure 5C). In baseline scenario A, inbreeding reached 0.70% (95% CI [0.64%, 0.76%]) in year 20. The polled selection conventional breeding scenarios, B, C, and D resulted in a similar 20-year inbreeding level of 0.70–0.76% (95% CI [0.64%, 0.82%], *P* ≥ 0.05 compared to A). The addition of gene editing the top 1% of seedstock bull calves per year to the polled preference mating schemes (B_1% and C_1%) increased 20-year inbreeding to 0.85% (95% CI [0.79%, 0.91%], *P* ≤ 0.05 compared to A). However, the addition of gene editing in all other scenarios (B_10%, C_10%, D_1%, and D_10%) resulted in similar 20-year inbreeding levels (*P* ≥ 0.05 compared to A). The inbreeding at year 20 based on an estimation of $\frac{1}{8N_{\text{m}}}+\frac{1}{8N_{\text{f}}}$ (*N*_*m*_ is number of mothers, *N*_F_ is number of fathers) was 0.22% for all scenarios, which is lower than that calculated by the simulation program due to the fact that the number of parents does not consider the relatedness between the seedstock animals as our simulation did. This in turn was higher than the inbreeding of 0.15% which would be expected under random mating.

#### Genetic Gain ($JapOx)

Baseline scenario A, which placed no selection pressure on polled, resulted in a 20-year $JapOx value of $226 (95% CI [$224, $228]; Figure 5E). Selection of polled sires in the conventional breeding scenarios (B, C, and D) decreased the 20-year $JapOx value by $32 (95% CI [$28, $37]), $28 (95% CI [$24, $33]), and $48 (95% CI [$43, $52]), respectively (*P* ≤ 0.05 compared to A).

The polled preference conventional breeding scenarios B and C’s 20-year $JapOx values were $194 (95% CI [$192, $196]) and $198 (95% CI [$196, $200], *P* ≤ 0.05 compared to B), respectively (Figure 5E). The addition of gene editing the top 1% of seedstock bull calves per year to these mating schemes (B_1% and C-1%) significantly increased the 20-year $JapOx value by $22 (95% CI [$17, $26], *P* ≤ 0.05 compared to B) and $17 (95% CI [$12, $21], *P* ≤ 0.05 compared to C, *P* ≥ 0.05 compared to B_1%), respectively. Moreover, the addition of gene editing the top 10% of seedstock bull calves per year (B_10% and C-10%) further increased the 20-year $JapOx value by $31 (95% CI [$26, $36], *P* ≤ 0.05 compared to B) and $29 (95% CI [$25, $34], *P* ≤ 0.05 compared to C, *P* ≥ 0.05 compared to B_10%), respectively. In fact, the 20-year $JapOx values in scenario B_10% ($225, 95% CI [$223, $227]) and C_10% ($227, 95% CI [$225, $229]) were not significantly different (*P* ≥ 0.9) from the baseline scenario A (Figure 5E).

The obligatory polled conventional breeding scenario D slowed the rate of genetic gain the most, resulting in a $178 (95% CI [$176, $180]) 20-year $JapOx value. The addition of gene editing the top 1 and 10% of seedstock bull calves per year to this scheme (D_1% and D_10%) significantly increased the 20-year $JapOx value by $10 (95% CI [$6, $14]) and $15 (95% CI [$11, $19]), respectively (*P* ≤ 0.05 compared to D). The 20-year $JapOx value in D_10% was similar ($193, 95% CI [$191, $195], *P* ≥ 0.05) to the polled preference conventional breeding scenario B.

Overall, the addition of gene editing the top 1 and 10% of seedstock bull calves per year resulted in significantly faster (*P* ≤ 0.05) rates of genetic gain compared to the conventional breeding scenarios (B, C, and D) of each polled mating scheme (Figure 5E).

#### Number and Genotype of Sires Used Per Year

**FIGURE 7:**
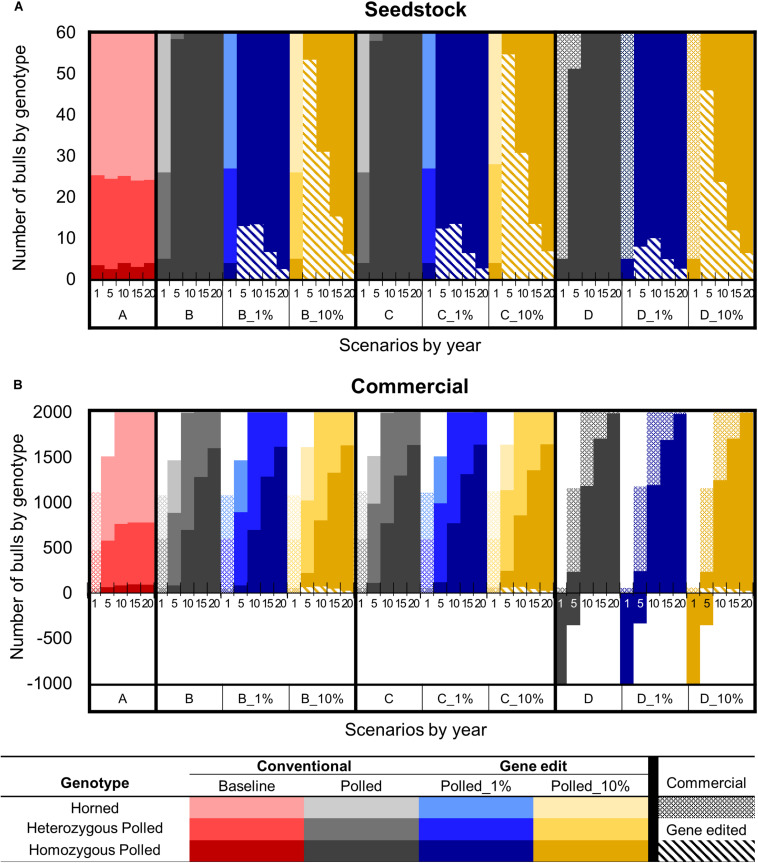
Effect of each scenario on the number of sires used by genotype in year 1, 5, 10, 15, and 20 for the seedstock **(A)** and commercial **(B)** populations. Genotypes are represented by a color scale from darkest (homozygous polled) to lightest (horned). Bulls produced via natural mating and sourced from the seedstock or commercial population are represented by solid or dotted bars, respectively. Gene edited seedstock sourced bulls are represented by diagonal hatched bars. Solid bars below the *x*-axis represent the deficit of bulls necessary to breed all females in the scenario.

In year one of all scenarios, there were ∼5 (6%) seedstock homozygous polled sires used for breeding out of the 60 total sires (Figure 7A). Baseline scenario A and polled preference schemes B and C also used ∼22 (36%) heterozygous polled and ∼35 (58%) horned seedstock sires. In contrast, due to the limited number of seedstock homozygous polled sires, ∼55 commercial homozygous polled bulls were needed to supplement the supply of seedstock bulls in year 1 of obligatory polled scheme D.

Baseline scenario A resulted in little change in genotype availability/use of seedstock sires throughout all 20 years. In contrast, by year 5 the polled preference conventional breeding scenarios (B and C) resulted in 56 (95% CI [52, 60], *P* ≤ 0.05) more homozygous polled sires used for breeding compared to A and 0 horned sires were used. Additionally, by year 10 all sires used for breeding were homozygous polled. However, the addition of gene editing the top 1 and 10% of seedstock bull calves per year to these polled preference schemes resulted in all sires used for breeding being homozygous polled by year 5. The greatest number of gene edited sires used for breeding was 15 (95% CI [14, 16]) around year 5 in scenarios B_1% and C_1%, whereas 55 (95% CI [53, 56], *P* ≤ 0.05 compared to B_1% and C_1%) were used around year 10 in scenarios B_10% and C_10% (Figure 7A).

Interestingly, the addition of gene editing the top 1% of seedstock bull calves per year to obligatory scheme D resulted in 5 (95% CI [2, 7], *P* ≤ 0.05) fewer gene edited sires being used for breeding in year 5 compared to B_1% and C_1%. Additionally, D_10% also resulted in 9 (95% CI [5, 11], *P* ≤ 0.05) fewer gene edited sires being used for breeding in year 10 compared to B_10% and C_10%. Fewer gene edited sires were used in obligatory scheme D because there were more conventionally bred homozygous polled sires available due to the mating scheme design.

There was a sequential drop in the number of gene edited sires used in all of the 1 and 10% gene editing scenarios after 5 and 10 years, respectively. The majority of sires used for breeding in these scenarios were either the offspring of gene edited sires from the previous years or produced via conventional breeding of parents both carrying the *POLLED* allele. In fact, by year 20 in the 1 and 10% gene editing scenarios for all the polled mating schemes (B, C, and D), only 3 (95% CI [2, 4]) and 7 (95% CI [6, 8], *P* ≤ 0.05) of the sires used for breeding were homozygous polled via gene editing, respectively (Figure 7A).

### Commercial Population

#### *HORNED* Allele Frequency

To model the current Australian Brahman population, all scenarios started with a *HORNED* allele frequency of 80% (Figure 5B). In baseline scenario A, which placed no selection pressure on polled, the commercial population *HORNED* allele frequency remained at a similar level throughout all 20 years. Consequently, after 20 years of baseline scenario A the commercial population was estimated to consist of only 5% homozygous polled, 34% heterozygous polled and a majority (61%) of horned animals (Figure 6B). In contrast, selection of polled sires in the conventional breeding scenarios (B, C, and D) decreased the 20-year *HORNED* allele frequency by 48.6% (95% CI [47%, 50%]), 49.1% (95% CI [48%, 50%]), and 70.0% (95% CI [69%, 71%]), respectively (*P* ≤ 0.05 compared to A; Figure 5B).

The polled preference conventional breeding scenarios B and C’s 20-year *HORNED* allele frequencies were 29.4% (95% CI [29% 30%], *P* ≤ 0.05 compared to A) and 28.9% (95% CI [28%, 29%], *P* ≤ 0.05 compared to A, *P* ≥ 0.05 compared to B), respectively (Figure 5B). As a result, after 20 years both polled preference scenario B and C’s commercial populations were estimated to be ∼50% homozygous polled and less than 10% horned (Figure 6B).

The addition of gene editing the top 1% of seedstock bull calves per year (B_1% and C-1%) resulted in a similar 20-year *HORNED* allele frequency (*P* ≥ 0.9) compared to conventional breeding scenarios, B and C, respectively. In contrast, the addition of gene editing the top 10% of seedstock bull calves per year (B_10% and C-10%) resulted in a 1.2% (95% CI [0.0%, 2.4%], *P* ≥ 0.05 compared to B) and 1.5% (95% CI [0.3%, 2.7%], *P* ≤ 0.05 compared to C, *P* ≥ 0.05 compared to B_10%) lower 20-year *HORNED* allele frequency, respectively (Figure 5B). These slightly lower 20-year *HORNED* allele frequencies of scenarios B_10% and C_10% were estimated to translate to approximately 2% more homozygous polled animals in year 20 (Figure 6B).

The obligatory polled scheme D resulted in the lowest 20-year *HORNED* allele frequency of 8.0% (95% CI [7.5%, 8.5%]; Figure 5B). In scheme D only homozygous polled sires were used, thus conventional breeding and the addition of gene editing the top 1 and 10% of seedstock bull calves per year resulted in the same (*P* = 1) rapid change in *HORNED* allele frequency. Additionally, scheme D was estimated to result in the highest percentage of homozygous polled animals (84%) in year 20 (Figure 6B).

#### Inbreeding

The commercial population inbreeding remained below 0.02% in all scenarios (Figure 5D). In baseline scenario A, inbreeding reached 0.005% (95% CI [0.0039%, 0.0053%]) in year 20. All of the preferential polled scenarios, including the addition of gene editing 1 and 10% of the seedstock bull calves per year, (B, B_1%, B_10%, C, C_1%, and C_10%) resulted in similar (*P* ≥ 0.05 compared to A) 20-year inbreeding levels of 0.005% (95% CI [0.0039%, 0.0063%]). All of the obligatory polled scenarios, including the addition of gene editing 1 and 10% of the seedstock bull calves per year, (D, D_1% and D_10%) resulted in a significantly higher 20-year inbreeding level of approximately 0.012% (95% CI [0.0105%, 0.0124%], *P* ≤ 0.05 compared to A). These values were slightly higher than the 0.006% 20-year inbreeding value estimated from only the number of parents in the population, which in turn was similar to the inbreeding value expected under random mating.

### Number and Genotype of Sires Used Per Year

#### Years 1–9

In year 1 of all scenarios, only commercial base population bulls were available for mating because the seedstock herds had not yet started supplying bulls to the commercial herds (Figure 7B). Baseline scenario A and polled preference schemes B and C used ∼1,100 commercial bulls (58% horned, 38% heterozygous polled, and 4% homozygous polled), which was a sufficient number to breed all mature commercial females in year 1. In contrast, obligatory polled scheme D only used ∼60 commercial bulls (100% homozygous polled) to breed in year 1. Consequently, there was a deficit of ∼1,000 bulls (i.e., ∼35,000 commercial cows were left open) in year 1 of obligatory polled scheme D.

After year 1, baseline scenario A and polled preference schemes B and C, only used bulls sourced from the seedstock population. In contrast, due to the limited number of homozygous polled sires, commercial bulls were needed to supplement the supply of seedstock bulls for all 20 years in obligatory polled scheme D. In this scheme, using a majority of commercial (∼900) and several seedstock (∼240) homozygous polled bulls, still resulted in a deficit of ∼350 bulls in year 5 (Figure 7B).

#### Years 10–20

After 10 years of baseline scenario A there was little change in genotype availability of sires, resulting in only 88 (95% CI [68, 108]) homozygous polled sires (4%) used for breeding compared to 1,217 (95% CI [1,203, 1,230]) horned (60%). In contrast, the polled conventional breeding scenarios (B, C, and D) resulted in 612 (95% CI [566, 658]), 685 (95% CI [639, 731]), and 1,912 (95% CI [1,866, 1,958]) more homozygous polled sires used for breeding, respectively (*P* ≤ 0.05 compared to A).

In year 10, the polled preference conventional breeding scenarios B and C used 700 (95% CI [680, 720], *P* ≤ 0.05 compared to A) and 773 (95% CI [753, 793], *P* ≤ 0.05 compared to A and B) homozygous polled sires for breeding, respectively. The addition of gene editing the top 1% of seedstock bull calves per year (B_1% and C_1%) resulted in a similar number (*P* = 1 compared to B and C, respectively) of homozygous polled sires used for breeding in year 10. In contrast, the addition of gene editing the top 10% of seedstock bull calves per year (B_10% and C-10%) resulted in 107 (95% CI [60, 153], *P* ≤ 0.05 compared to B, *P* ≥ 0.05 compared to C) and 89 (95% CI [43, 135], *P* ≤ 0.05 compared to C) more homozygous polled sires used for breeding in year 10, respectively (Figure 7B).

The obligatory polled scheme D clearly used the greatest number of homozygous polled sires (2000, 95% CI [1980, 2020], *P* ≤ 0.05 compared to A, B, and C) in year 10 and thereafter. However, ∼38% (821, 95% CI [811, 830]) of the bulls used for breeding in year 10 of the obligatory polled conventional breeding scenario D were commercial, which on average had lower genetic merit than seedstock bulls. The addition of gene editing the top 1% of seedstock bull calves per year to this scheme (D_1%) resulted in a similar number of commercial bulls used for breeding in year 10 (806, 95% CI [796, 815], *P* ≥ 0.05 compared to D). In contrast, the addition of gene editing the top 10% of seedstock bull calves per year to this scheme (D_10%) significantly decreased the number of commercial bulls used for breeding in year 10 by 71 (95% CI [49, 94], *P* ≤ 0.05 compared to D).

In year 10, the polled selection scenarios that included gene editing the top 10% of seedstock bull calves per year (B_10%, C_10%, and D_10%), used 73 (95% CI [69, 77], *P* ≤ 0.05 compared to B_1%, C_1%, and D_1%) gene edited seedstock bulls, which was ∼4% of the total bulls used for breeding. By year 20 of these scenarios (B_10%, C_10%, and D_10%), only ∼1% (26, 95% CI [24, 29], *P* ≤ 0.05 compared to B_1%, C_1%, and D_1%) of the bulls used for breeding were gene edited seedstock bulls (Figure 7B).

### Number of Animals Sold for Beef Per Year

**FIGURE 8:**
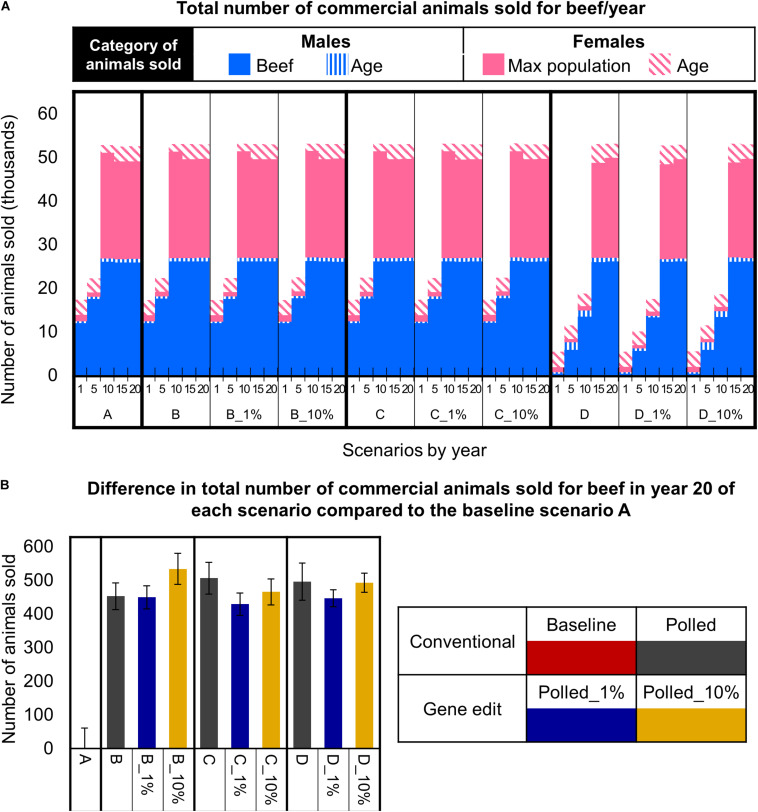
Effect of each scenario on the total number of commercial animals sold for beef. **(A)** Number and category of animals sold in year 1, 5, 10, 15, and 20 for each scenario. **(B)** The difference in the number of animals sold in year 20 for each polled mating scenario compared to the baseline A scenario. Error bars represent SEM.

In the baseline scenario A and polled preference schemes B and C, the maximum commercial cow population size was reached in year 10. Thereafter, in these schemes there were ∼61,000 cows bred per year (due to 3-year mating age limit), all mature females were mated, and ∼53,000 animals (i.e., steers, age-culled males and females, and population size-culled females) were sold for beef per year (Figure 8A). Strikingly, in year 10 and thereafter the polled preference conventional breeding scenarios B and C resulted in significantly more animals sold for beef per year (20-year = 470 more, 95% CI [202, 762], *P* ≤ 0.05) compared to baseline scenario A (Figure 7B).

In contrast, due to the limited number of homozygous polled sires available (Figure 7B), the obligatory polled scheme D reached the maximum commercial cow population size approximately 5 years later than schemes A, B, and C (Figure 8A). Consequently, scheme D resulted in only ∼18,000 (95% CI [18,082, 18,476], *P* ≤ 0.05 compared to A) animals sold for beef in year 10 (Figure 8A). However, in year 15 and thereafter, even the obligatory polled scheme D resulted in a significantly higher number of animals sold for beef per year (20-year = 470 more, 95% CI [172, 805], *P* ≤ 0.05) compared to baseline scenario A (Figure 8B).

The addition of gene editing the top 1 and 10% of seedstock bull calves per year to the polled mating schemes (B, C, and D) resulted in similar (*P* ≥ 0.3) numbers of total animals sold for beef per year for each respective polled mating scheme (Figure 8A). Overall, all polled mating scenarios resulted in significantly more (*P* ≤ 0.05) animals sold for beef in year 20 than baseline scenario A (Figure 8B). This difference was due to decreased dehorning-related calf loss as a direct outcome of producing fewer horned calves.

## Discussion

Animal Health Australia (AHA) held an open public consultation and found that issues related to “pain relief for surgical procedures” (e.g., dehorning) were the most controversial for the Australian public (Animal Health Australia [AHA], 2014b). All animal welfare and animal rights groups, and some academic groups pushed to mandate pain relief irrespective of the age of the animal, while many producer groups (including major national and northern Australian cattle producer groups) advised that mandating pain relief at any age is impractical. Therefore, there is an urgent need to reduce the need for dehorning. Additionally, dehorning in northern Australia has considerable economic costs for producers due to the ∼2% mortality rate associated with the process (Bunter et al., 2013). Fortunately, increasing polled, or hornless, genetics presents an opportunity to address this major animal welfare concern while eliminating a major post-weaning mortality factor. However, there are trade-offs to consider.

### Current Polled Status of Australian Brahmans

We found that the *POLLED* allele frequency is much higher in the Australian Brahman population (20%, Figure 4) compared to <3% in the United States dairy population (Spurlock et al., 2014; Mueller et al., 2019). This supports the finding of Lyons and Randhawa (2020). Interestingly, the average genetic merit of Australian Brahman horned bulls was similar compared to heterozygous polled bulls. In contrast, the average genetic merit of Australian Brahman homozygous polled bulls was significantly less (AUD $6 $JapOx) than both horned and heterozygous polled bulls. However, this difference is much less drastic than between United States dairy homozygous polled and horned bulls (∼USD $100 Lifetime Net Merit index, NM$) (Mueller et al., 2019).

Neither $JapOx nor NM$ incorporate polled directly into the selection index, although new horned calves were penalized with a 2% dehorning mortality rate in the current simulation study. Cole (2015) found that the inclusion of an economic weight on polled based on the cost of dehorning (including reduced calf health and increased calf mortality) had no effect on allele frequency in the United States Holstein population. Rather, the primary determinant of success with breeding for polled was the initial allele frequency in the population being studied. Adding a polled status weighting to $JapOx in the current study would not have altered the number of polled bulls available in scenarios B, C, and D and thus would not have altered the rate of *HORNED* allele frequency change in those scenarios. However, it would have increased the $JapOx value of polled sires which would have accelerated the decrease of the *HORNED* allele frequency in the baseline scenario A if the weighting exceeded the genetic merit differential between horned and homozygous polled bulls.

It should be noted that the average genetic merit values used in this study may not be completely representative of the Australian Brahman population as producers are unlikely to have sent in samples from horned animals for polled DNA testing. The data did suggest a slight over representation of heterozygotes, although the observed frequencies were not significantly different from those expected under Hardy–Weinberg equilibrium. However, if homozygous polled animals have a different genetic merit value profile in reality than were used in this study, then the results would alter accordingly.

### Mating Schemes

Several mating schemes were modeled in the current study to represent a business-as-usual scenario and potential situations that might arise if some, or drastic (i.e., prohibited production of horned calves) market pressure is placed on the Australian beef industry to eliminate dehorning. In our simulation results, both the seedstock and commercial population results for each scenario followed similar patterns. Although each scenario had a greater impact on the seedstock population due to its smaller size, an important measure of a seedstock population is its gene flow into the larger commercial population. Therefore, the following discussion will focus primarily on the commercial population results.

#### Baseline (A)

Baseline scenario A placed no selection pressure on polled genetics, which represents industry practices where breeders are solely focused on improving genetic merit ($JapOx). This scenario resulted in little change in *HORNED* allele frequency (Figures 5A,B) or genotypes of sires used for breeding (Figure 6). As mentioned, there is a larger proportion of polled Australian Brahman sires compared to United States dairy sires. Irrespective, the baseline scenario demonstrated that, as with the United States dairy industry (Cole, 2015; Mueller et al., 2019), strong selection pressure will need to be placed on polled genetics in order to meaningfully increase the number of polled Australian Brahman cattle.

The baseline scenario A commercial population reached a 20-year $JapOx value of $160 ($8/year; Figure 5F). The baseline scenario commercial population gain of $8 per year is higher than the current rate of gain for the Australian Brahman population (Australian Brahman Breeder’s Association [ABBA], 2018). One reason for this high rate of gain is that this simulation assumed that TBV for $JapOx were known (i.e., breeding value accuracy = 1). Additionally, no regard was given to practical considerations and other selection criteria such as temperament, phenotype, and structure, which would reduce the rate of gain, and also influence selection for poll/edited animals and therefore decrease the rate of actual population change in polled. Conversely, no selection was placed on females in this simulation. Voluntary culling of horned cows would be another way to reduce the *HORNED* allele frequency, although it may limit the number of cows actually available to breed in some scenarios and make little difference in others. For example, in scenario D, due to population size, there is no involuntary culling of cows until year 15, and by that time there are no horned animals remaining to cull.

Baseline scenario A demonstrates the trade-offs associated with the business-as-usual situation. While scenario A resulted in one of the fastest rates of genetic gain, this scenario also used the most horned bulls for breeding throughout all 20 simulated years (Figures 7A,B) and consequently produced the most horned calves. Due to the northern Australian environment and extensive management practices there is a ∼2% mortality rate associated with dehorning (Bunter et al., 2013). This dehorning mortality rate was included in our model and resulted in scenario A having the smallest number of total animals sold for beef in year 20, which is a major opportunity cost associated with continuing to produce horned calves (Figure 8B).

#### Polled Preference (B and C)

The polled preference scenarios were chosen to represent an intermediate situation where some producers prioritize using polled sires (scenario B) or choose to only use homozygous polled sires (scenario C) while other producers continue to select sires based solely on $JapOx. This situation may arise if AHA were to implement the public suggestion of “mandating pain relief irrespective of the age of the animal” for surgical procedures, such as dehorning (Animal Health Australia [AHA], 2014b). Both scenario B and C resulted in a similar decrease of commercial *HORNED* allele frequency to ∼30% by year 20.

Interestingly, the Australian Brahman homozygous polled-only preference scenario C results contrast with the same scenario modeled for the United States dairy population. In the United States dairy simulations, scenario C (i.e., preferentially using homozygous polled sires) did not significantly reduce the *HORNED* allele frequency because there were not enough homozygous polled Holstein sires available (only 1% of total sires) for scenario C to be successful. In contrast, there is a larger proportion of Australian Brahman homozygous polled sires available (6% of total sires), so C was the optimal conventional breeding scenario for the Brahman population in terms of substantially decreasing *HORNED* allele frequency while sustaining the rate of genetic gain. This simulation finding is in agreement with Lyons and Randhawa (2020). When polled and horned cohorts’ BREEDPLAN EBVs were compared, they concluded, “An increased prevalence of the polled condition within herd or industry-wide would not be expected to have a measurable negative influence on production, carcass, fertility and behavior traits” (Lyons and Randhawa, 2020).

As demonstrated in polled preference scenarios, B and C, Australian Brahman producers currently have genetic options available to proactively address this animal welfare concern before it becomes a legal requirement. In our simulation there was a significant decrease ($1.30/year) in the rate of genetic gain in conventional breeding scenario B and C compared to the baseline scenario A. However, after 10 years of both B and C there was a benefit in that these scenarios had less dehorning-related calf loss resulting in over 200 more steers sold for beef annually as compared to scenario A.

Additionally, producing high-genetic-merit polled sires through gene editing could mitigate the economic trade-off of the slower rate of genetic gain associated with increasing *POLLED* allele frequency through conventional breeding methods. Consistent with United States dairy findings (Mueller et al., 2019), the scenarios that placed strong selection pressure on polled (scenarios B and C) combined with the use of gene editing, were the optimum solution for decreasing the number of horned calves, while still maintaining the rate of genetic gain.

In the United States dairy population, the addition of gene editing only the top 1% of genetic merit bull calves per year to the polled preference schemes was sufficient to maintain the same or better rate of genetic gain compared to baseline A, while significantly decreasing horned to less than 10% (Mueller et al., 2019). Due to the widespread use of AI, a single dairy sire can have an immense impact on the whole population, thus only a small number of elite dairy sires need to be gene edited to see population level results.

In contrast, due to natural mating limits, individual Australian Brahman gene edited bulls did not have an extensive impact on the whole population. While the addition of gene editing the top 1% of Australian Brahman seedstock bull calves per year did significantly increase the rate of genetic gain ($0.80/year) compared to the conventional breeding scenarios, scenarios B_1% and C_1% still resulted in significantly slower ($0.50/year) rates of genetic gain compared to baseline A. However, the addition of gene editing the top 10% of Australian Brahman seedstock bull calves per year did result in similar rates of genetic gain to baseline A. Therefore, the overall optimal Australian Brahman scenario based on maintaining the rate of gene gain and rapidly decreasing *HORNED* allele frequency was C_10%.

#### Obligatory Polled (D)

Obligatory polled scheme D modeled a case where market expectations or government policies force the Australian beef industry to completely ban dehorning, as previously advocated for by animal rights groups (Animal Health Australia [AHA], 2014b). This situation would require either the exclusive use of homozygous polled sires to ensure no horned progeny were produced or leaving all horns intact. Although, it is worth noting that a requirement for live export is cattle being polled or dehorned such that each horn stump is less than 12 cm in length and fully healed (LiveCorp-Australia, 2001).

We chose to model the use of commercial homozygous polled sires to supplement the supply of seedstock bulls when necessary. This model could be achieved by separating and leaving intact (i.e., not castrating) any commercial bulls without horns during the first calf gathering and processing. These phenotypically hornless commercial bulls could then be genomically tested to determine their polled genotype. The confirmed homozygous polled commercial bulls could be retained or sold for breeding while the heterozygous polled commercial bulls could be castrated and sold for beef as usual.

The obligatory polled conventional breeding scenario D, resulted in the lowest 20-year *HORNED* allele frequency of less than 10%, but scenario D also had the slowest rate of genetic gain of $5.50/year, compared to baseline scenario A of $8.00/year. Although there was a significant difference between the Australian Brahman obligatory polled conventional breeding scenario D and baseline A rates of genetic gain (∼$2.50/year), the difference was much less drastic than between the similar United States dairy scenarios (∼$34.00/year) (Mueller et al., 2019). Additionally, unlike United States dairy simulations, the obligatory polled scheme D did not increase inbreeding above acceptable levels. Instead the primary issue for Australian Brahman’s in this scenario was not having a sufficient number of homozygous polled bulls to breed all commercial females via natural service, even when supplementing the seedstock supply of bulls with bulls sourced from the commercial sector.

In this simulation, once all the seedstock and commercial homozygous polled bulls reached their maximum 35 matings per year, the remaining females were left open. This model was chosen to demonstrate the current deficit of homozygous polled Australian Brahman bulls that would be needed to make this obligatory polled scheme D feasible. A more realistic alternative would be to use homozygous polled bulls from other less tropically adapted breeds. However, this alternative could result in increased animal welfare issues and higher levels of mortality due to ill-adapted sires and their resultant progeny (Bunter et al., 2013).

Using gene editing to rapidly produce homozygous polled sires could be a tool to mitigate the negative impacts of a situation where market expectations or government policies force the exclusive use of homozygous polled sires, as demonstrated in obligatory polled scheme D. The addition of gene editing the top 1 and 10% of seedstock bull calves per year to this scheme (D_1% and D_10%) significantly increased the 20-year commercial $JapOx value by $8 ($0.40/year) and $15 ($0.75/year), respectively, compared to conventional breeding scenario D. Gene editing enables the production of high-genetic-merit homozygous polled sires in only one generation as opposed to the several generations of back-crossing necessary to recover the high-genetic-merit when the *POLLED* allele is introgressed via traditional crossbreeding (Tan et al., 2012). Additionally, the immediate increase in the availability of homozygous polled sires enabled by gene editing resulted in more homozygous polled progeny in the following years. As a result, 15 (D_1%) and 71 (D_10%) fewer bulls sourced from the commercial sector were needed to breed commercial cows in year 10 compared to conventional breeding scenario D. This is important because the commercial bulls on average had lower genetic merit compared to the bulls sourced from the seedstock population.

### Considerations for Increasing *POLLED* Allele Frequency in the Northern Australian Beef Cattle Population

While few studies have investigated polled beef breeding schemes, multiple studies have simulated a variety of conventional breeding and genomic selection schemes to increase *POLLED* allele frequency in the United States and European dairy populations, while minimizing negative impacts on inbreeding and genetic merit (Spurlock et al., 2014; Cole, 2015; Gaspa et al., 2015; Windig et al., 2015; Scheper et al., 2016; Segelke et al., 2016). Similar to the current study, these simulations revealed that a fast transition to a completely polled population, as was modelled in obligatory polled scheme D, substantially reduced the rate of genetic gain. The dairy industry has the advantage of being able to use AI sires, and females are also often genotyped. This means that there can be a higher intensity of selection for polled on the male side, and some selection on the female side (Gaspa et al., 2015), as compared to the extensive northern Australian beef cattle situation.

#### Inbreeding

In the commercial population of baseline scenario A and all other scenarios inbreeding increased less than 0.005% per year, and never exceeded 0.02% (Figures 5C,D). This level of inbreeding has been found to have relatively minor effects on traits of economic or biological significance in tropical beef cattle (Burrow, 1998). In these Australian Brahman scenarios inbreeding was not a major concern largely due to the sires’ limited influence by natural mating. One natural service bull can have a maximum of ∼100 progeny throughout its lifetime. In contrast, inbreeding is a primary concern in the United States dairy population due to widespread use of AI enabling an individual sire to produce thousands of progeny and have an extensive influence on the population (Mueller et al., 2019). It should be noted that the inbreeding estimate in the current simulation assumed that all base population animals were initially unrelated, which is unlikely to be valid in a commercial setting. If in reality Australian Brahman polled animals are more related to each other than horned animals, then the rate of inbreeding would increase faster than occurred in this simulation. Alternatively, if Australian Brahman polled animals are less related to horned animals, then the rate of inbreeding may increase more slowly than in our simulation.

#### Potential to Use Gene Editing to Produce High-Genetic-Merit Polled Sires

In our simulations there were ∼1000 seedstock bull calves born each year, so ∼10 and ∼100 calves were gene edited to be homozygous polled per year in the _1% and _10% scenarios, respectively. The current northern Australian cattle population of ∼6 million breeding-age females requires ∼171,500 total bulls assuming 35 matings per bull per year (Ashton et al., 2016). On average, a bull is used for breeding for 3 years, so the seedstock population needs to produce ∼57,000 bulls each year. Therefore, gene editing 1% and 10% of the northern Australian bull calves per year would be ∼570 and 5,700 calves, respectively. Given this scale, gene editing 10% of the bull calves produced per year may be impractical, irrespective of economic feasibility questions.

The gene editing scheme modeled in this paper (Figure 1), based on the proposed Kasinathan et al. (2015) elite sire production system, is different from gene editing scenarios modeled in other simulations. In this paper fetal tissue from the next generation of yet-to-be born bulls was genomically screened and selected, edited, and then successfully cloned such that the editing step added an additional 3–5 months to produce a gene edited, homozygous polled bull (Figure 1). While this method is technically possible (Kasinathan et al., 2015), if instead samples for editing are being collected after animals are born, then this would introduce a time lag of 2–3 years into the editing simulations modeled in this paper.

In contrast, Bastiaansen et al. (2018) modeled gene editing of a recessive allele at the one-cell zygote stage, and gene edited all zygotes from either 0, 10, or 100% of matings from genomically-selected elite parents. They then modeled various gene editing success and survival rates, based on the low efficiencies of gene editing reported in the literature (Tan et al., 2016). When they modeled 100% accuracy and survival, they observed a strong favorable impact of gene editing on decreasing the time to fixation for the desired allele, compared to genomic selection alone. However, the efficiencies of a gene knock-in via template-guided techniques, like introgressing the P_*C*_ allele, is much lower than 100% in zygotes. When they modeled a 4% gene editing efficiency, this had a major impact on the number of editing procedures and on the loss in selection response (Bastiaansen et al., 2018).

Another simulation study modeled promotion of alleles by genome editing (PAGE) to improve quantitative traits, by selecting and gene editing the best animals based on their breeding value (Jenko et al., 2015). Even though the Jenko et al. (2015) study did not model a recessive monogenic trait (e.g., polled), their simulation was more similar to the editing system modeled in this study, as compared to Bastiaansen et al. (2018). The editing system modeled in this Australian Brahman study also avoided the challenge of mosaicism because the genotype of the edited cell line could be confirmed before cloning (Bishop and Van Eenennaam, 2020). However, when using this approach, a cloning step is required to produce an animal from the edited cell line. SCNT cloning is prohibited in several European countries (van der Berg et al., 2020). Additionally, cloning itself is also inefficient with only 6–15% of transferred embryos resulting in a viable calf (Chavatte-Palmer et al., 2012).

Irrespective of the approach employed, the use of gene editing will require an efficient *in vitro* embryo production and ET program (McFarlane et al., 2019; Bishop and Van Eenennaam, 2020). In this Australian Brahman simulation study, the gene edited bulls only produced progeny via natural service (i.e., 35 matings per bull per year). We modeled solely natural mating because currently reproductive tools are scarcely used in this population (Meat and Livestock Australia Limited [MLA], 2015). However, this is unlikely to be the situation with valuable gene edited bulls. It is more probable that a high-genetic-merit homozygous polled sire would be used for AI or *in vitro* embryo production followed by ET, in the seedstock sector. This system would amplify the reach of each gene edited bull using well-proven advanced reproductive technologies and enable these bulls to produce hundreds or even thousands of progeny, and thus have a greater impact on the whole population. This is perhaps a more realistic scenario than what was modeled in this paper, but our objective was to compare the current system with and without gene editing, rather than additionally increase the intensity of selection, which would have further accelerated the rate of genetic gain in the gene editing scenarios.

### Regulatory Considerations of Gene Editing

In 2019 the Australian government announced they will not regulate the use of gene editing techniques in plants, animals and human cell lines that do not introduce new genetic material (Mallapaty, 2019). However, gene editing technologies that use a template-guided technique (e.g., the *POLLED* allele) or that insert other genetic material into the cell, will continue to be regulated by the Office of the Gene Technology Regulator (OGTR) as Genetically Modified Organisms (GMO). This ruling may make it cost-prohibitive to use gene editing to introduce the *POLLED* allele into multiple Australian beef bulls and cattle breeding programs, due to the expense and uncertainty associated with the GMO regulatory approval process (Van Eenennaam et al., 2019). It should be noted that an on-target integration of donor template plasmid backbone occurred in one of two edited alleles (Norris et al., 2020; Young et al., 2020) in research cattle that were gene edited to carry the P_*C*_ allele (Carlson et al., 2016). To avoid this, researchers have transitioned to plasmid-free single stranded DNA (ssDNA) repair templates for small genomic alterations, such as the P_*C*_ allelic introgression, which have a significantly reduced frequency of unintended genomic integration (McFarlane et al., 2019). Irrespective, this ssDNA would still be considered a template-guided technique and would trigger GMO regulation in Australia.

Currently, the global regulatory status of gene edited animals is uncertain, and is the primary concern for further investment and development of gene edited animals (Zhao et al., 2019; van der Berg et al., 2020). Editing that does not introduce novel recombinant DNA sequences would not be treated differently to conventional breeding in some countries, e.g., Argentina (Whelan and Lema, 2015) and Brazil (United States Department of Agriculture [USDA], 2019), whereas in the United States intentional genomic alteration in animals are subject to mandatory premarket regulation as a new veterinary drug (Solomon, 2020). In the European Union, animal cloning is currently prohibited which would preclude SCNT-enabled editing approaches as modeled in this paper, and in 2018 the European Court of Justice ruled that organisms obtained by directed mutagenesis, including gene editing, will be regulated as GMOs (van der Berg et al., 2020). Conversely, in Canada traditional mutagenesis is not regulated unless it produces a novel trait. In that country it is the novelty of product that triggers regulation, not the method used to produce that novel trait. And finally, New Zealand plans to regulate all gene edited animals, irrespective of the nature of the edit, as GMOs (Fritsche et al., 2018).

Many of these regulatory decisions have been made in the absence of engagement with the scientific community, industry, stakeholders or publics (Bruce and Bruce, 2019). In particular, the judgment of the European Court of Justice to subject gene edited organisms to GMO regulations, but to exempt organisms produced using the older less precise process of mutagenesis breeding defied risk proportionality and reasonableness, to the dismay of European scientists (Hundleby and Harwood, 2019). It remains to be seen whether animal breeding companies can successfully overcome the technical and regulatory challenges that must be faced to employ gene editing for the genetic improvement of commercial livestock.

## Data Availability Statement

The collated Australian Brahman genotype and EBV dataset that underlies several of the simulation parameters used for this study is not readily available due to privacy restrictions. Requests to access the datasets should be directed to GeneSeek^®^ and the Australian Brahman Breeders Association Limited. Python programs and notebooks used for simulation and analysis of gene editing in cattle breeding programs can be found at https://github.com/wintermind/gene-editing.

## Author Contributions

JC and MM authored and modified the “geneedit” software. MM performed all the simulations and data analysis with additional input from JC, DJ, and AVE. DJ facilitated obtaining access to northern Australian cattle data. NC and IR provided and collated genotype data and breeding values for Australian Brahman cattle. MM wrote the manuscript with input from JC, DJ, and AVE. All authors reviewed and approved the final manuscript.

## Conflict of Interest

The authors declare that the research was conducted in the absence of any commercial or financial relationships that could be construed as a potential conflict of interest.

## Appendix

### Appendix 1: Information Needed to Reproduce the Simulations Used in This Study

**APPENDIX TABLE 1:** Simulation parameters.

Software parameter	Definition	Value used for each mating scheme				
		A	B	C		D
**Program control parameters**												

debug	Show or hide debugging messages	TRUE				
rng_seed^1^	Value used to seed the random number generator	Time + PID				
embryo_inbreeding	Save file with embryo inbreeding (makes large files)	FALSE				
history_freq	Frequency with which history files are written (end of the simulation or every generation)	‘end’				
show_recessives	Show recessive frequencies after each round of simulation	FALSE				
check_all_parms	Perform a formal check on all parameters	TRUE				
show_disposals	Print the frequency of disposals by reason	TRUE				
filetag	Label with which to append filenames for tracking different scenarios	‘scenario_name’				
nucleus_filetag	Label with which to append filenames for tracking different scenarios	‘scenario_name_nuc’				
generations	How long to run the simulation	20				

**Base population (multiplier) parameters**												

base_herds	Number of herds in the population	200				
base_bulls	Initial number of founder bulls in the population	1500				
base_max_ bull_age	Maximum age of base population bulls	4				
base_cows	Number of cows in the base population	35000				
base_max_ cow_age	Maximum age of base population cows	10				
genetic_sd	Additive genetic SD of the simulated trait	34				
cow_mean	Average base population cow TBV	0				
bull_diff	Differential between base cows and bulls, in genetic SD	1				
base_polled	Genotype of polled animals in the base population (‘homo’| ‘het’| ‘both’)	‘both’				
polled_parms	Proportion of polled bulls total, proportion of PP, and proportion of Pp bulls	[0.33, 0.08, 0.92]				
polled_diff	Differential between Pp and pp bulls, in genetic SD	[0.00,				
	Differential between PP and pp bulls, in genetic SD	0.16]				

**Scenario (multiplier) parameters**												

bull_unique	Create unique bull portfolios for each herd	TRUE				
service_bulls	Number of herd bulls to use in each herd each generation	10				
max_matings	The maximum number of matings permitted for each bull (5% of cows for dairy AI)	35				
bull_criterion	How should the bulls be picked for portfolios?	‘random’	‘polled’	‘polled’	‘polled’	
bull_copies	Genotype of polled bulls selected for mating (0 = PP, 4 = PP or Pp)	n/a	4	0	0	
bull_deficit	Manner of handling too few polled bulls for matings: ‘use_ horned’ or ‘use_ mult’ or ‘no_ limit’ or ‘open’	‘open’	‘use_ horned’	‘use_ horned’	‘use_ mult’	
bull_mating_age	The minimum age for bulls to breed cows (set to 1 for dairy)	2				
cow_mating_age	The minimum age for cows to be bred and have a calf in the same generation (set to 1 for dairy)	3				
calf_loss	Rate of calfhood mortality	0.13				
dehorning_loss	Rate of mortality during dehorning	0.02				
harvest_beef	Percentage of (multiplier) males that are harvested at ∼1 year of age for Beef	1				
max_bulls	Maximum number of live bulls to keep each generation	2000				
max_bull_age	Age of bulls beyond which they’re culled	4				
max_cows	Maximum number of live cows to keep each generation	100000				
max_cow_age	Age of cows beyond which they’re culled	10				
flambda	Decrease in economic merit per 1% increase in inbreeding	0				
carrier_penalty	Penalize carriers for carrying a copy of an undesirable allele (True), or not (False)	FALSE				
percent	Proportion of bulls to use in the truncation mating scenario	n/a				

**Base population (nucleus) parameters**												

use_nucleus	Create and use nucleus herds to propagate elite genetics	TRUE				
nucleus_base_herds	Number of herds in the population	10				
nucleus_base_bulls	Initial number of founder bulls in the population	600				
nucleus_base_max_bull_age	Maximum age of base population bulls	4				
nucleus_base_cows	Number of cows in the base population	1500				
nucleus_base_max_cow_age	Maximum age of base population cows	10				
nucleus_genetic_sd	Additive genetic SD of the simulated trait	34				
nucleus_cow_mean	Average base population cow TBV	34				
nucleus_bull_diff	Differential between base cows and bulls, in genetic SD	1				

**Scenario (nucleus) parameters**												

nucleus_service_bulls	Number of herd bulls to use in each herd each generation	6				
nucleus_max_matings	The maximum number of matings permitted for each bull (5% of cows for dairy AI)	35				
nucleus_calf_loss	Rate of calfhood mortality	0.08				
nucleus_dehorning_loss	Rate of mortality during dehorning	0.02				
nucleus_harvest_beef	Percentage of (multiplier) males that are harvested at ∼1 year of age for Beef	0				
nucleus_bulls_to_move	The maximum number of bulls to move from nucleus herds to the multiplier herds each year	1000				
nucleus_max_bulls	Maximum number of live bulls to keep each generation	60				
nucleus_max_bull_age	Age of bulls beyond which they’re culled	4				
nucleus_max_cows	Maximum number of live cows to keep each generation	3000				
nucleus_max_cow_age	Age of cows beyond which they’re culled	10				

**Gene editing parameters**												

edit_prop	Percent of bulls and cows edited in different scenarios	(bulls)	0	0	1	10	0	1	10	0	1	10
	(cows)	0				
edit_type^2^	Technologies used for gene editing	‘C’				
edit_trials	The number of attempts to edit an embryo successfully (−1 = repeat until success)	−1				
embryo_trials	The number of attempts to transfer an edited embryo successfully (−1 = repeat until success)	−1				

*^1^Time system clock time when the simulation is submitted, PID process identification reported by the operating system, ^2^C CRISPR-Cas9.*
